# Report of simultaneous measles outbreaks in two different health regions in Portugal, February to May 2017: lessons learnt and upcoming challenges

**DOI:** 10.2807/1560-7917.ES.2019.24.3.1800026

**Published:** 2019-01-17

**Authors:** Gonçalo Figueiredo Augusto, Andreia Silva, Natália Pereira, Teresa Fernandes, Ana Leça, Paula Valente, Etelvina Calé, Bárbara Andreia Aguiar, António Martins, Paula Palminha, Elsa Vinagre, Rita Cordeiro, Sílvia Lopo, Paulo Jorge Nogueira

**Affiliations:** 1 Directorate-General of Health, Lisbon, Portugal; 2 National Institute of Health Dr Ricardo Jorge, Lisbon, Portugal; 3 Laboratory of Biomathematics, Faculty of Medicine, University of Lisbon, Lisbon, Portugal

**Keywords:** measles, outbreak, Portugal, B3, epidemiology, elimination, vaccination, immunity, immunisation, vaccination coverage

## Abstract

In Portugal, measles vaccination coverage and population immunity are high, and no endemic measles cases had been reported since 2004. The World Health Organization classified measles as eliminated in the country in 2015 and 2016, based on data from the previous 3 years. However, in a context of increasing incidence in several European countries in 2016 and 2017, Portugal experienced two simultaneous measles outbreaks with a total of 27 laboratory-confirmed cases (0.3 cases/100,000 population) in two health regions between February and May 2017. Nineteen cases (70.1%) were adults, of whom 12 were healthcare workers. Overall, 17 cases (63.0%) were not vaccinated, of whom five were infants younger than 12 months of age. One unvaccinated teenager died. Genotype B3 was identified in 14 cases from both regions. Measles virus sequencing identified different possible origins of the virus in each region affected. Although measles transmission was stopped in less than 2 months from the first case being notified, these outbreaks represent an opportunity to reinforce awareness of measles diagnosis. We highlight the intensity of the control measures taken and their impact on the rapid control of the outbreaks and also the fact that high vaccination coverage was crucial to stop transmission.

## Introduction

Measles is one of the most highly contagious infectious human diseases and can cause serious illness, lifelong complications and death. The widespread use of safe and cost-effective measles vaccines in national immunisation programmes globally has resulted in a steep decrease in measles cases and deaths worldwide [1]. Following the 2010 decision by the Member States in the World Health Organization (WHO) European Region to initiate the process of verifying elimination, the European Regional Verification Commission for Measles and Rubella Elimination was established in 2011 [1]. In this context, the Global Measles and Rubella Strategic Plan 2012–2020 and the European Vaccine Action Plan 2015–2020 both include measles elimination as a main objective [2,3].

**Figure 1:**
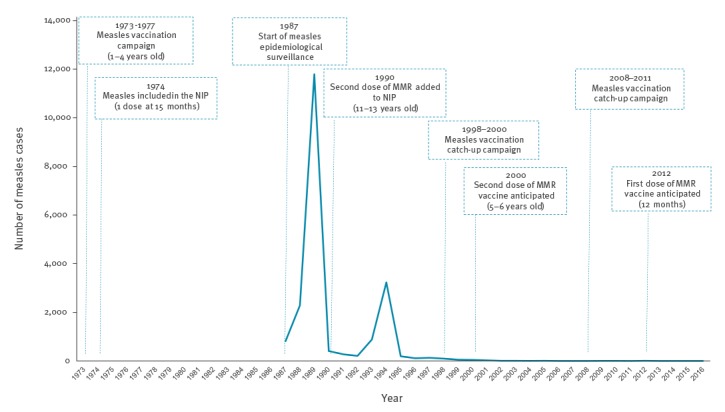
Evolution of measles vaccination strategy and number of measles cases in Portugal, 1973–2016 (n = 20,589)

MMR: measles-mumps-rubella; NIP: National Immunisation Programme.

Source: Directorate-General of Health (Direção-Geral da Saúde, DGS).

Currently, in accordance with the Portuguese National Immunisation Programme (NIP), two doses of MMR vaccine are recommended for children (at 12 months and 5 years of age) [4]. Due to consistent and sustained high immunisation coverage against measles (> 95%), the number of measles cases has declined dramatically over the past two decades (Figure 1). The last major measles outbreaks took place in 1987–89 and 1993–94, and the last reported suspected endemic measles cases in Portugal were reported in 2003. Thus, WHO Europe classified measles as eliminated in the country in 2015 and 2016, based on data from the previous 3 years [5].

In a context of increasing number of outbreaks in European countries [6], with 30 European Union (EU)/European Economic Area (EEA) countries reporting 5,881 cases between 1 March 2016 and 28 February 2017 [7], two measles outbreaks were detected in Portugal at the beginning of 2017 [8]. The first outbreak was identified in the Algarve health region (southern Portugal) and another outbreak was identified in the Lisbon and Tagus Valley health region. The latter lasted until May 2017 and, overall, 27 confirmed cases were notified to health authorities.

The aim of this article is to describe, beyond the previous rapid communication [8] and in further detail, the two measles outbreaks that occurred in the Algarve and Lisbon and Tagus Valley health regions between February and May 2017, the control measures taken, their impacts in the community, the workforce involved, the challenges faced in contact tracing and the lessons learnt.

## Methods

### Measles epidemiological surveillance

Physicians who suspect a measles case are expected to report to local public health authorities and to collect samples to send to the WHO-certified national reference laboratory for measles and rubella, the National Institute of Health (Instituto Nacional de Saúde Doutor Ricardo Jorge, INSA) [9,10]. Notification to public health authorities is currently done electronically through the National System for Epidemiological Surveillance (Sistema Nacional de Vigilância Epidemiológica, SINAVE), which records clinical and laboratory notifications. Likewise, INSA also reports laboratory results electronically through SINAVE. Following each clinical notification, an automatic email alert is generated for local, regional and national public health authorities. Local public health authorities are responsible for undertaking epidemiological investigation and implementation of immediate control measures for each suspected case identified. All cases described in these outbreaks were notified through SINAVE. The first cases identified in both transmission chains were first notified by the laboratory, while the others were primarily notified by clinicians. For all confirmed cases, there was a clinical and a laboratory notification.

### Case definition and classification

The measles case definition and classification used during these outbreaks meet the criteria of the European Union case definition [11] and have already been described in the previous rapid communication [8].

### Epidemiological investigation

Local public health units were responsible for undertaking epidemiological investigation and implementation of control measures for each suspected measles case identified. Regional public health departments coordinated those investigations and communicated with the national level: INSA and DGS. For each suspected measles case, extensive contact tracing was carried out, which allowed, for confirmed cases, the identification of earlier cases who had not yet been diagnosed or notified. Epidemiological investigations also made it possible to identify and document clear links between confirmed cases in both transmission chains.

### Laboratory investigation

Laboratory investigation was carried out by INSA. Laboratory tests included serum IgG and IgM measurements, or measles nucleic acid detection or measles virus isolation in oral fluids, throat swabs or urine. Genetic characterisation was carried out in all measles-RNA-positive cases. Genotype was determined by sequence analysis of the 450 nt that code the C-terminal of the nucleoprotein (N) according to WHO protocol [12].

## Outbreak description

From 1 January until 30 June 2017, 243 suspected measles cases were reported in Portugal, of which 222 were laboratory investigated. During this period, 27 cases were confirmed, 5 were possible, and 211 were discarded. Among confirmed cases, two imported measles cases were identified in the North and Alentejo health regions, which corresponded to isolated cases with no epidemiological or genotypic links to the cases in the two outbreaks described in this paper.

**Figure 2:**
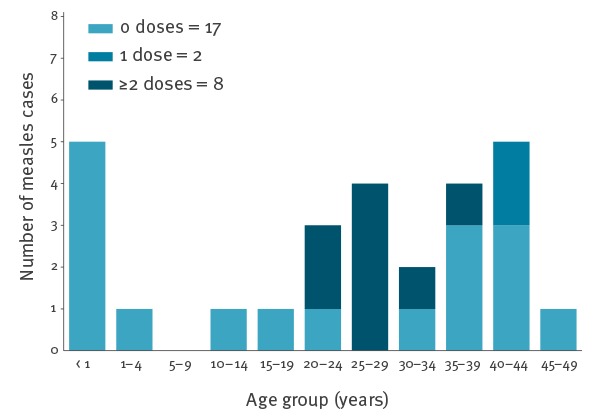
Measles cases by vaccination status, Algarve and Lisbon and Tagus Valley health regions, Portugal, February–May 2017 (n = 27)

Source: National System for Epidemiological Surveillance (Sistema Nacional de Vigilância Epidemiológica, SINAVE) / Directorate-General of Health (Direção-Geral da Saúde, DGS).

**Figure 3:**
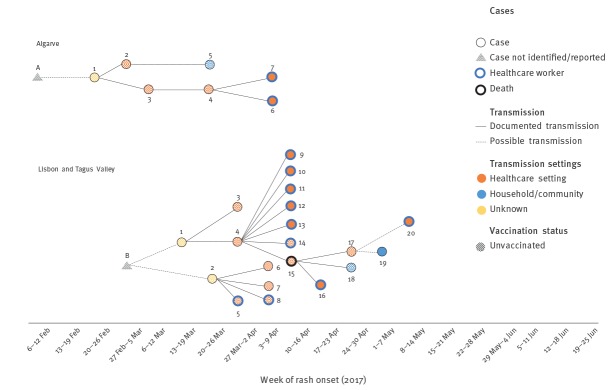
Measles transmission chains, Algarve and Lisbon and Tagus Valley health regions, by week of rash onset, Portugal, February–May 2017 (n = 27)

Source: Directorate-General of Health (Direção-Geral da Saúde, DGS).

Overall, the two outbreaks included 27 confirmed cases in two health regions: Algarve (7 cases, 1.58/100,000 population) and Lisbon and Tagus Valley (20 cases, 0.55/100,000 population) (Figure 2). Of the 27 confirmed cases, 17 were unvaccinated, 12 were healthcare workers, and one unvaccinated teenager died (Figure 3).

### Algarve outbreak

The outbreak in the Algarve health region was notified to health authorities on 30 March.

Overall, this transmission chain comprised seven confirmed cases (Figure 3; Table). Cases 1, 2, 3 and 4 were infants younger than 1 year and therefore were not yet vaccinated with the measles-mumps-rubella (MMR) vaccine. Case 5 was an unvaccinated adult. Only Cases 6 and 7 had been vaccinated with two doses of the MMR vaccine, with the second dose given more than 10 years. Six of these cases acquired measles in a healthcare setting, including two healthcare workers.

**Table:** Characteristics of measles cases, Algarve and Lisbon and Tagus Valley health regions, Portugal, February–May 2017 (n = 27)

Region	Case	Age group (years)	Sex	Rash onset	MMR doses	Last dose	Hospitalisation	Complications	Genotype
Algarve	1	< 1	M	23 Feb	0	NA	Yes	Pneumonia	NK
	2	< 1	F	5 Mar	0	NA	Yes		NK
	3	< 1	M	11 Mar	0	NA	Yes		NK
	4	< 1	M	21 Mar	0	NA	Yes		NK
	5	19–25	M	21 Mar	0	NA	Yes		NK
	6	26–35	F	6 Apr	2	1991	No		B3
	7	19–25	F	8 Apr	2	2003	No		B3
Lisbon and Tagus Valley	1	36–45	F	17 Mar	0	NA	Yes	Pneumonia	NK
	2	36–45	F	22 Mar	0	NA	Yes		NK
	3	36–45	F	27 Mar	0	NA	Yes		NK
	4	< 1	M	30 Mar	0	NA	Yes		B3
	5	36–45	F	1 Apr	0	NA	No		NK
	6	36–45	F	3 Apr	0	NA	No	Diarrhoea	NK
	7	36–45	F	4 Apr	0	NA	Yes		NK
	8	26–35	M	4 Apr	0	NA	No		B3
	9	26–35	F	11 Apr	2	1995	No		NK
	10	36–45	F	12 Apr	1	1976	No		B3
	11	19–25	F	12 Apr	2	2007	No		B3
	12	36–45	F	13 Apr	1	1977	No		B3
	13	19–25	F	13 Apr	2	2002	No		B3
	14	36–45	F	13 Apr	0	NA	Yes	Diarrhoea	B3
	15	1–18	F	13 Apr	0	NA	Yes	Pneumonia	B3
	16	26–35	M	23 Apr	3	1996	No		B3
	17	< 1	M	24 Apr	0	NA	No		NK
	18	1–18	F	25 Apr	0	NA	Yes		B3
	19	19–25	F	5 May	2	2001	No		B3
	20	19–25	M	13 May	2	2003	No		B3

F: female; M: male; MMR: measles-mumps-rubella; NA: not applicable; NK: unknown.

Source: National System for Epidemiological Surveillance (Sistema Nacional de Vigilância Epidemiológica, SINAVE) / Directorate-General of Health (Direção-Geral da Saúde, DGS).

As Case 1 had not travelled abroad, the most likely hypothesis, in a context of measles elimination with high population immunity and in a popular European touristic destination, is that an unknown case (Case A) who acquired measles abroad, came into contact with Case 1 around week 6 2017. However, Case A was neither diagnosed nor reported to health authorities.

### Lisbon and Tagus Valley outbreak

The outbreak in the Lisbon and Tagus Valley health region was notified to health authorities on 6 April.

This transmission chain comprised 20 confirmed cases (Figure 3; Table), including two infants, two adolescents and 16 adults. Of the 20 cases, 10 were healthcare workers, 12 were unvaccinated, 8 were hospitalised, and one died.

Cases 1 and 2 did not report recent travel abroad and did not have contact with each other. The fact that both had disease onset within 5 days suggests that they may have acquired measles from a common source. As in the Algarve health region, the most likely hypothesis is that an unknown measles case who acquired measles abroad (Case B) came into contact with these two cases in different settings. Case B was neither diagnosed nor reported to health authorities.

### Characteristics of cases

The median age of the 27 confirmed cases was 25 years (range: 0–45 years). Most confirmed cases (n = 19) occurred in adults (≥ 18 years), two cases were adolescents, and six cases occurred in infants under 15 months of age (Table). Twelve cases were healthcare workers.

Of the 27 cases, 17 had not been previously vaccinated, while the remaining cases had documented evidence of one (n = 2), or two or more doses (n = 8) of a measles-containing vaccine, either single or combined (Table). Of the 10 cases who were previously vaccinated, nine were healthcare workers (Table).

Among the unvaccinated cases (n = 17), five were infants under 12 months of age and thus too young to be vaccinated, one was a 13-month-old infant, two were adolescents, and the remaining nine cases were adults (Table).

Two of the 12 healthcare workers had received one dose and seven two or more doses of measles-containing vaccine; three healthcare workers had not been previously vaccinated (Table).

## Laboratory results

Up to 30 June, samples from 222 suspected measles cases were sent to INSA for laboratory investigation, of which 27 cases related to these outbreaks were laboratory-confirmed measles cases. Eleven cases were confirmed by using PCR testing of oral fluids or urine specimens, while other 11 cases were confirmed by detection of measles-specific IgM antibodies in serum; in four cases, both IgM and PCR positive test results were reported, and one case was confirmed through elevation of IgM levels in a pair of titres.

For one of the 15 PCR-confirmed cases, the genotype could not be identified, because of low number of copies. In the remaining 14 cases, the sequence was identified as the B3 measles virus, which is the same genotype detected in other outbreaks in Europe in 2016 and 2017, including Belgium and Italy [13,14]. However, through the phylogenetic analysis of the measles virus, it was possible to identify two possible different origins.

In the Algarve health region, the identified sequences were phylogenetically similar to the virus type circulating in Germany in 2016 and 2017, which suggests that Case A (neither identified nor reported) could have travelled from Germany by the end of January 2017 and come into contact with with Case 1 in the Algarve health region (Figure 3).

In the Lisbon and Tagus Valley health region, the 12 identified sequences were all the same, even though not all epidemiological links in this cluster were clearly documented. Theses sequences were phylogenetically identical to the virus type circulating in France and Italy in 2016 and 2017, which suggests that Case B could have travelled from France or Italy in end of February 2017 and come into contact with Cases 1 and 2 in the Lisbon and the Tagus Valley health region (Figure 3).

## Control measures

The increasing number of measles cases reported in several European countries in 2016 and early 2017 led the Directorate-General of Health (Direção-Geral da Saúde, DGS) to issue an alert to healthcare services, followed by recommendations and guidelines about diagnosis, early detection and response to measles cases, within the scope of the National Measles Elimination Programme [10]. After the identification of the first measles case, a contingency plan was implemented, which included four main axes: (i) containment, prevention and control; (ii) training; (iii) information sources and (iv) communication.

A specific algorithm for early detection of measles was also created within the National Health Service contact centre (SNS 24) for triage of suspected cases by phone.

**Figure 4:**
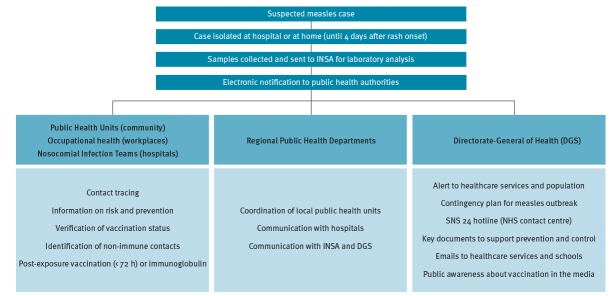
Control measures during the measles outbreak, Algarve and Lisbon and Tagus Valley health regions, Portugal, February–May 2017

INSA: National Institute of Health; NHS: National Health Service.

In these outbreaks, all reported suspected measles cases were investigated and control measures were promptly implemented at the local level to contain transmission (Figure 4). According to national guidelines, when a suspected measles case is identified by a physician, the patient should be immediately isolated (at the hospital or at home) until 4 days after rash onset. Simultaneously, samples must be collected and sent to INSA for laboratory investigation and the case must be notified to public health authorities (Figure 4).

SNS 24 is the National Health Service hotline, giving health advice and referring patients to healthcare if needed.

Several teams undertook extensive and rapid contact tracing for all measles cases. While public health units mainly conducted contact tracing in the community, occupational health teams and nosocomial infections teams undertook contact tracing at workplaces and hospitals, respectively.

Contacts and relatives of cases were informed about measles transmission, risk and prevention, and their vaccination status was assessed. When unvaccinated contacts were identified, these were vaccinated whether the case was confirmed or not. Thus, this strategy was useful to enhance vaccination in the community. Close contacts of confirmed cases were offered either post-exposure vaccination or immunoglobulin.

Besides interviewing patients, hospital staff and family members, public health authorities had to liaise with airline companies and foreign public health authorities, as one confirmed case had travelled abroad during the incubation period (Case 16 from the Lisbon and Tagus Valley chain of trainsmission).

Epidemiological investigations and control measures were complemented with broader public health actions. Those were carried out by DGS and included the dissemination of key documents to support prevention and control measures, for example, posters about clinical features of measles, guidelines, epidemiological bulletins and background materials for healthcare services [15-17].

Additionally, DGS sent several emails to reinforce information about measles risk and prevention, targeting healthcare workers and schools, and raised public awareness about the importance of vaccination through numerous reports in national media. Following these outbreaks, and in the scope of the National Measles Elimination Programme, DGS also set up a measles vaccination catch-up campaign [18].

## Discussion

These outbreaks were the largest to have occurred in Portugal since 1993–94. The fact that the country had not had any endemic measles cases for more than a decade represented a challenge for health services in terms of diagnosis and sampling of all suspected cases identified. Despite extensive contact tracing and investigation of the possible source of infection for each measles case, this was the first time, since 2004, that outbreaks had occurred without identification of the imported primary cases. However, these two outbreaks were also an opportunity to increase measles diagnosis awareness among healthcare workers, as evidenced by the identification and investigation of more than 200 suspected measles cases between March and June 2017. Additionally, 1,200 and 1,600 contacts of suspected cases were investigated by local public health authorities in the Algarve and Lisbon and Tagus Valley health regions, respectively. Identifying close contacts represents a challenge and additional effort for health authorities, as health services need to be adjusted when faced with an outbreak, including human resource management (for the rapid identification of contacts, vaccination post-exposure in the first 72 hours, administration of immunoglobulin to susceptible persons), financial resources (strengthening vaccine stock, ensuring immunoglobulin availability). To this end, health service resilience is essential for quick control of the outbreak.

Immunity against measles is high among the Portuguese population, due either to the free circulation of the virus until 1972 or to sustained vaccination since 1973. In contrast to other European countries, Portugal has achieved sustained high immunisation coverage against measles. Vaccination coverage of two MMR doses in the population below 18 years of age has been at least 95% for more than two decades [19]. The third National Serological Survey (2015–16) showed a proportion of immune individuals of 94.2% in the general population [20], confirming the results of the second survey (2001–02) [21]. Results by age group show that those > 55 years and < 10 years were the age groups with the highest immunity (measles-specific IgG antibodies ≥ 200 mUI/mL) [20]. People aged between 20 and 29 years had the lowest immunity against measles [20]. In fact, most cases of the outbreaks presented here occurred in young adults who were either unvaccinated or had been vaccinated more than 10 years previously. This seems to be a possible consequence of lower antibody levels due to the absence of natural boosters (no circulation of the virus in the community) when the virus was circulating. In fact, the majority of vaccinated cases were healthcare workers who came into contact with measles cases. None of the vaccinated cases in both outbreaks was hospitalised or had complications.

The fact that healthcare settings were the main route of transmission in both outbreaks represented a challenge because any person in hospital environment, regardless of their role, can be affected, since measles is highly contagious and persists in the environment for up to 2 hours, requiring immediate implementation of control measures [22,23]. The NIP recommends two doses of MMR for healthcare workers in Portugal, but four cases were either unvaccinated or incompletely vaccinated. Therefore, verification of healthcare workers’ immunisation status and vaccination of unvaccinated or non-immune individuals is demonstrated to be a critical component of the National Measles Elimination Programme. Given the high risk of exposure and transmission that healthcare workers face, it is not surprising that even some who had received two doses of MMR vaccine became infected with measles virus. However, it is important to note that vaccinated healthcare workers experienced mild measles infection, did not need hospitalisation and did not transmit the disease.

These outbreaks and all the communication actions taken made healthcare workers and the general public aware that measles is still a threat and a serious disease, which can cause hospitalisations and deaths. As a result, not only during but also after the outbreak, demand for vaccination increased (data not shown).

Measures were taken to reinforce vaccination in communities where MMR coverage was lower than 95%. A national catch-up campaign was set-up covering the following groups: (i) children and adolescents younger than 18 years (recommended schedule of two doses), with focus on pockets of susceptible population; (ii) healthcare workers (complete two doses for those who have never had the disease); and (iii) adults (≥ 18 years old) born in or after 1970 (one dose for those who have never been vaccinated and never had the disease), with focus on those aged 18–30 years.

Although the primary cases in both outbreaks could not be identified, measles sequencing was crucial to document the introduction of two different B3 genotypes in Portugal, in the context of increasing numbers of outbreaks in European countries since 2016 [7]. Given the epidemiological situation in other European countries and the increasing popularity of Portugal as a travel destination, Portuguese public health authorities should remain alert and strengthen epidemiological surveillance to avoid future outbreaks.

### Lessons learnt

Continuous maintenance of high vaccination coverage rates is critical to stopping transmission chains and controlling measles outbreaks. To this end, it is important not only to notify unvaccinated people but also to implement innovative strategies to raise awareness among the population.

It was found that persons adequately vaccinated and with a high level of exposure when providing healthcare to cases developed measles; however, they presented a mild clinical picture.

It was verified that the cases occurring in vaccinated persons did not generate secondary cases, which is important for the prevention of transmission chains in health services.

The resilience of health services to measles outbreaks is a challenge which needs to be given particular attention by policymakers because of the high cost of resources involved in control measures.

In terms of the international epidemiological context, EU/EEA countries should maintain a high alert level.

Portugal is strongly committed to meeting the criteria defined by the WHO to maintain the status of measles elimination.

### Conclusion

High vaccination coverage, and early and effective implementation of control measures contributed to the rapid interruption of measles transmission in both regions affected. Although Portugal has been successful in meeting WHO’s objective of eliminating measles in the European Region, this disease is a challenge that requires a coordinated effort from all European countries due to the high risk of measles importation. Sustained high vaccination coverage, effective epidemiological surveillance and early implementation of control measures are critical to quickly contain outbreaks such as the one described here, and to interrupt virus circulation. These outbreaks represent an opportunity to strengthen the existing National Measles Elimination Programme and to enhance vaccination both in the community and among healthcare workers.
